# Evaluation of Wound Healing Properties of Grape Seed, Sesame, and Fenugreek Oils

**DOI:** 10.1155/2016/7965689

**Published:** 2016-11-20

**Authors:** Dorsaf Moalla Rekik, Sameh Ben Khedir, Kamilia Ksouda Moalla, Naziha Grati Kammoun, Tarek Rebai, Zouheir Sahnoun

**Affiliations:** ^1^Laboratory of Pharmacology, Faculty of Medicine of Sfax, University of Sfax, Sfax, Tunisia; ^2^Laboratories of Histology and Embryology, Faculty of Medicine of Sfax, University of Sfax, Sfax, Tunisia; ^3^Olivier Institute, BP 1087, 3000 Sfax, Tunisia

## Abstract

*Background*. Medicinal plants have proved at all times to be a powerful remedy for health care. Accordingly, grape seed, sesame, and fenugreek extracted oils with pharmacological properties are investigated as wound treatments. This study assesses the potential of our oils for healing wounds induced on rats.* Methods*. Phytochemical analyses of oils have involved: quality value, polyphenol, chlorophylls, carotene, and fatty acids. Antibacterial activity was carried out. Antioxidant activity was evaluated: the scavenging effect on DPPH radicals, the reducing power, and *β*-carotene discoloration. Uniform wound excision was induced on rats dorsum randomly divided into five groups: groups treated with “*CICAFLORA*®” and tested oils and untreated one. The posthealing biopsies were histologically assessed.* Results*. Wound biopsies treated with oils showed the best tissue regeneration compared to control groups. Groups treated with our oils and “*CICAFLORA*” had higher wound contraction percentage. Polyunsaturated fatty acids in oils act as inflammatory mediators increasing neovascularization, extracellular remodeling, migration, and cell differentiation. Wound healing effect was attributed to antibacterial and antioxidant synergy.* Conclusion*. According to findings, oils showed better activity in wound healing compared to “*CICAFLORA*” due to a phytoconstituents synergy. However, clinical trials on humans are necessary to confirm efficacy on human pathology.

## 1. Background

Wounds regardless of their types and causes are common diseases that constitute a major problem of public health at the global level and mainly in countries in the process of development. However, despite the impressive progress in modern medicine, drugs dispensed to treat the skin represent 3% of the intended ones and are not yet really effective.

Various medicinal plants, mainly their oils, have always been used to treat different kinds of wounds. The literature presents various herbal formulations and natural extracts with several phytochemical compounds (vitamins, phenols, sterols, etc.), in healing plants for the application of wound care. A whole list of those medicinal plants are traditionally used in folk medicine, including our three selected plants, grape seed, fenugreek, and sesame, which are investigated in this study in order to explore their phytochemical compositions, to evaluate their wound healing effect and to better explain the mechanism on wound healing.

*Trigonella foenum graecum *L. (Fenugreek) is an annual herb that belongs to the* Fabaceae* family, commonly used in oriental countries as a spice in food preparations. The seeds are reported to have nutritive and restorative properties and can stimulate digestive processes [1]; moreover, they are used as a traditional remedy for the treatment of diabetes [2].

*Sesamum indicum* L. (Sesame) is a pioneer cultivated self-pollinating annual plant, originating from Africa, belonging to the* Pedaliaceae *family. Sesame takes an important role in human nutrition and the seeds are essentially used for the production of oil.

The fruit is a micronized and oblong capsule containing numerous seeds [3]. This oilseed has numerous nutritional [4], ethnobotanical, pharmaceutical, and medical applications. In fact, it is laxative, emollient, and demulcent [5]. This urges us to further explore this oilseed and focus our concern on dermal repair, stressing our research on wound healing in particular.

*Vitis vinifera *L. (grapes) belongs to the* Vitaceae* family. Grapes have been a traditional treatment in Europe for thousands of years. The grape seed oil is rich in unsaturated fatty acids, especially linoleic acid [6] which constitutes a considerable proportion of the seed.

Wound healing, the significant concern in pathology like in postsurgeries, burns, and scars, is a dynamic and complex process that involves biochemical and physiological phenomena from inflammation to proliferation to remodeling, behaving in a harmonious way to ensure tissue repair [7]. During the inflammatory phase, this process is hampered by the production of a high level of free radicals. If not controlled by the antioxidative capacity of the host, the inhibition of both the cell migration and the proliferation takes place, which can damage the surrounding, wound tissue [8]. So, the present study evaluates the wound healing effect of three medicinal plants previously referred to (the fenugreek seed oil, the sesame seed oil, and the grape seed oil) via the exploration of phytochemical composition and the different parameters of antibacterial and antioxidant activities to explain the crucial mechanism of wound healing.

## 2. Methods

### 2.1. Plant Material and Reagents

#### 2.1.1. Reagents

The (DPPH) 1,1-diphenyl-2-picrylhydrazyl was purchased from Sigma Chemical Co. (St. Louis, MO, USA). Butylated-hydroxytoluen (BHT) and all the other chemicals were of analytical grade.

For the evaluation of the wound healing, “*CICAFLORA*” was used as a reference product. It consists in an emulsion oil-water that contains* Mimosa* as a main active component and is marketed in a cream form.

#### 2.1.2. Materials

The analysis of the methyl esters of fatty acids was made by chromatography in gas phase (C.P.G) by means of a UNICAM 610 chromatograph, equipped with a detector (FID) allowing the detection of compounds, a column (15 m in length and 0.22 mm in diameter) lined with a film (0.25 *μ*m thick) of a polar phase (50% cyanopropylmethyl and 50% phenylmethyl-polysiloxane), and an injector divisor. The detector was at a temperature of 250°C, the column at 180°C, and the injector at 220°C. The quantity of injected oil was 0.2 *μ*L.

### 2.2. Analytical Methods

#### 2.2.1. Phytochemical Analysis

*(1) Quality Value and Fatty Acids*

*(i) Peroxide Value*. The peroxide value of any oil is an important indicator of primary oxidation level according to ISO 3960/2001 method. The index of peroxide of a fatty acid is the number of milliequivalents of active oxygen contents in 1 kg of the product and oxidizing the iodine of potassium with the liberation of iodine and titration of this one by the thiosulfate of sodium.

*(ii) Acidity Value*. The acidity value indicates the content of free fatty acids present in the tested oils, expressed in oleic acid. It represents an important quality parameter for the commercial classification of the product according to ISO 660/2003 method. 5 g of oil was dissolved in 30 mL of equal volumes of ethanol/ether (1/1) neutralized. The free carboxylic functions were measured by a solution of potassium hydroxide in the presence of 1% phenolphthalein. The end of the experience is marked by the appearance of a pink color.

*(iii) The Saponification Value*. The saponification value is an indirect measure that allows classifying the oil according to the length of fatty acid chains; the criterion was bound to the molecular weight of fatty acids. This measure can turn out useful because this value gives an idea about the quality of oils according to ISO 3657/2002 method. The value of saponification represents the quantity in milligrams of necessary potassium hydroxide to transform the free fatty acids and the glycerides contained in 1 g of fat into soap and is determined by mixing a volume of oil with potassium hydroxide and titrated with hydrochloric acid.

*(iv) Specific Extinction Coefficient at 232 nm and 270 nm*. The determination of the UV-specific extinction values gives an approximation of the oxidation process in unsaturated oils [9] according to COI/T.20/Doc (No 19/Rev.2 of November 2008) method. 0.25 g of the oil was dissolved in 25 mL of cyclohexane. The absorbance of the solution of fatty oil was measured by UV/visible spectrophotometer at specific wave length of 270 nm. The extinction at 270 nm of a raw fat can give an idea about the level of secondary oxidation.

*(v) Free Fatty Acid Percentage*. The free fatty acid method, COI/T.20/Doc (No 34, November 2015), determines the free fatty acids in oils by chromatography in gas phase. The content of free fatty acids is expressed in acidity, calculated as the percentage of oleic acid. It consists in diluting 0.2 g of extracted oil in 3 mL hexane and 0.3 mL of methanolic potassium hydroxide. The reactive mixture is shaken in a hanging whirlpool for 2 min and then spin-dried. Thus, the upper phase contains the esters of fatty acids dissolved in the hexane and the lower phase is trained by the fraction of glycerin and the minor constituents of the blank oil.

*(2) Quantification of Polyphenols*. The content of total phenols of the various samples is determined according to the method described by Tsai et al. [10]. In this method Folin-Ciocalteu reagent was added to the test oil. After agitation, a solution of washing soda is added. After 2 h of incubation, the absorbance is measured at 760 nm.

*(3) Chlorophylls and Carotene*. The contents of chlorophylls pigments and carotenoids in oils were calculated after the reading of the optical densities at 670 nm and 470 nm using coefficients of extinction for carotene and total chlorophylls [11].

#### 2.2.2. Antioxidant Activities

*(1) DPPH Free Radical-Scavenging Assay*. This assay has been used to investigate the scavenging activity of antioxidant compounds. In fact, DPPH is a stable free radical that can be reduced by a proton-donating substrate like an antioxidant, causing the discoloration of DPPH and reduction of the absorbance at 517 nm.

The DPPH free radical-scavenging potential of the three studied oils was determined according to the reports of Bersuder et al. [12]. Radical-scavenging activity was expressed as the inhibition percentage and was calculated using the equation of DPPH radical scavenging activity.

(1) DPPH  radical-scavenging  activity%=Acontrol−AsampleAcontrol×100.

*A*_control_ is the absorbance of the control reaction and *A*_sample_ is the absorbance of oils/standard BHT samples. The IC50 value (mg sample/mL) is the effective concentration at which the DPPH radicals are scavenged by 50%. The test was carried out in duplicate.

*(2) Ferric Reducing Antioxidant Power FRAP*. The ability of the oils (0.06 mg/mL to 1 mg/mL) to reduce iron (III) was determined according to the method of Yildirim et al. [13]. The IC50 value (mg sample/mL) is the effective concentration at which the absorbance is 0.5 for the reducing power. BHT is used for comparison and all data values are the mean of duplicate analysis.

*(3) β-Carotene Bleaching Assay*. This spectrophotometric technique in the ultraviolet ray was developed by Marco [14] and then slightly modified by Miller [15]. It consists in a measurement at 470 nm. The discoloration of *β*-carotene results from an oxidation by the linoleic acid.

#### 2.2.3. Antibacterial Activity

*(1) Microbial Strains*. The antimicrobial activity of the studied oils was evaluated using a range of laboratory control stains: two Gram-positive bacteria:* Bacillus subtilis* (JN 934392) and* Staphylococcus aureus* (ATCC 6538); two Gram-negative bacteria:* Escherichia coli* (ATCC 25922) and* Salmonella enteritidis* (ATCC 43972).

*(2) Determination of Antibacterial Activity by the Disc Diffusion Method*. The oils were tested for antibacterial activity by the method of disc diffusion according to the National Committee for Clinical Laboratory Standards (NCCLS, 2001) using suspension of the tested microorganisms. Mueller-Hinton agar (MHA), sterilized in a flask and cooled, was distributed to sterilized Petri dishes. The filter paper discs (6 mm in diameter) were individually impregnated with oil and then placed onto the agar plates which had previously been inoculated with the tested microorganisms. The Petri dishes were kept at 4°C for 2 h. The plates were inoculated with the bacteria and incubated at 37°C for 24 h. The diameters of all the inhibition zones were measured in millimeters. All the tests were performed in duplicate.

*(3) Determinations of the Minimum Inhibitory Concentration (MIC) and Minimum Bactericidal Concentration (MBC)*. The microdilution method was used to investigate the minimum inhibitory concentration (MIC) and the minimum bactericidal concentration (MBC) according to the National Committee for Clinical Laboratory Standards (NCCLS, 2001). All tests were performed in Mueller Hinton Broth (MHB). The oils were dissolved in 20% dimethylsulfoxide (DMSO) and then diluted from the highest concentration to the lowest one. A serial doubling dilution of the oils was prepared in a 96-well plate. Overnight broth cultures of each strain were prepared. Petri dishes were kept at 4°C for 2 h. Then, bacteria were incubated at 37°C for 24 h. The microbial growth was determined by absorbance at 600 nm using the universal microplate reader [16]. To evaluate MBC, broth from each well was taken and inoculated in Mueller Hinton Agar (MHA) at 37°C for 48 h for the bacteria.

*(4) MBC/MIC Ratio*. The MBC/MIC ratio [17] can give a clear idea about the effect of oils under study on bacteria. Indeed, if the ratio is higher than 4, the oil is said to be bacteriostatic and if the oil is endowed with a bactericide effect, the ratio is lower than 4.

#### 2.2.4. Wound Healing Activity Test

*(1) Experimental Animals*. Thirty Wistar male rats weighing 175 ± 3.98 g were used for the experiment. They were randomly housed in clean polyethylene cages individually under controlled conditions of 22–25°C, 60–70% relative humidity, and 12 hours of dark-light cycle with free access to water and food. Procedures and animal comfort were controlled by the International Guidelines for Animal Care.

*(2) Circular Excision Wound Model*. After anesthesia with ketamine (100 mg/kg body weight) by intramuscular injection, a full thickness of elliptic area of approximately 200 mm^2^ wound was induced on the shaved rats' dorsal interscapular region [18]. The day on which wound was created was considered as day 0 and all the wounds were covered with a gauze dressing and were treated until they completely healed.

*(3) Excision Wounds Treatment*. The rats were divided into five groups consisting of six rats each. Group number (I) was untreated and served as a control (the wounds were just cleaned with a physiologic saline). Group (II) was treated with fenugreek oil, group (III) with sesame oil, and group (IV) with grape seed oil and served as the test groups, while group (V) was treated with “*CICAFLORA*” cream and served as a standard reference (positive control).

After rinsing the wounds with the physiologic saline, the test samples (fenugreek oil, grape seed oil, and sesame oil) and the “*CICAFLORA*” cream were applied, in a fine layer covering all over the surface of the wound, every two days until the wound completely healed. So, the treatment was stopped when the wounds of any first group completely healed; then the rats were sacrificed and the granulation tissues were excised from animals. A part of wet tissue was fixed in formalin at 10% (v/v), embedded in paraffin, and presented for histological observation.

*(4) Wound Healing Evaluation Parameters*. To evaluate the process of wound healing for the 5 study groups, we relied on two clinical macroscopic criteria including the qualitative (color of wound) and the quantitative criteria (wound closure rate) and one microscopic criterion (histological evaluation).

*(5) Chromatic Study*. Superficial wounds tend to lighten from red to pale pink and become more homogeneous and more consistent in texture when they heal. The chromatic evaluation of the healing process was done through photography of wounds. This study consists of coding the wound of each rat: bright red for blood that covers the wound, dark red for coagulated blood in epidermis, red for granulation tissue, and finally pink for epithelialization phase [19].

*(6) Rate of Wound Closure and Epithelialization Time*. The rate of closure of each individual wound from both controls and the treated groups was used as an indicator of wound healing. A wound margin was traced after the wound incision using transparent paper and then the area was measured through the Mayrovitz rule [20]. Wound contraction was measured on the 3rd, 5th, 7th, 9th, and 10th days until complete wound healing and expressed in percentage of healed area. Wound contraction, the percentage of reduction of the original wound size, was calculated using the following expression:

(2) $$\begin{array}{c} Wound\;\;closure\left(\;\%\;\right)=\frac{\left(A_{T}-A_{D}\right)}{A_{T}}\times 100 \end{array}$$

The initial wound area on day 0 and the wound area on all the following days are represented as *A*_*T*_ and *A*_*D*_, respectively.

The epithelization period was considered as the number of required days to fall of scab without any residual raw wound [21].

*(7) Histological Examination*. All of the skin samples were fixed in 10% neutral buffered formalin. Following the fixation, 3 *μ*m sections of paraffin were perpendicularly made to the surface of skin including the whole thickness of skin. Serial sections were stained with hematoxylin-eosin (HE) [22] to show the morphology of tissues: organization, epithelial proliferation and granuloma tissue formation, collagenisation, newly capillaries formed, and scar formation in dermis.

The studied criteria in histopathological sections consisted in the reepithelialization, cornification of the epithelium, fibroblast, and collagen contents. Furthermore, histological biopsies examined for advanced tissue regeneration were characterized by the presence of well-organized stratum of both epidermis and derma.

#### 2.2.5. Statistical Analysis

Statistical analyses were performed using SPSS version 17 (SPSS Inc., Chicago, Il, USA).

Student test was applied to compare weights averages before and after treatment between oil-treated groups and both negative and positive control groups to detect ascertain significant differences. Nonparametric tests, Kruskal Wallis test, and Mann Whitney test were used to compare different groups. Raw data were shown with median IQR for each group. Differences were considered to be statistically significant at *p* < 0.05.

## 3. Results

### 3.1. Phytochemical Analysis

#### 3.1.1. Quality Value and Fatty Acids

Table 1 presents the values of acidity, specific extinction coefficient, peroxide, and the saponification.

Table 2 shows a different percentage of polyunsatured acids especially oleic, linoleic, and linolenic acids that reach together 89.37%, 82.5%, and 84.28%, respectively, for grape seed oil, sesame oil, and fenugreek oil.

#### 3.1.2. Quantitative Polyphenols, Chlorophylls, and Carotene

The quantitative dosage of total phenolic compounds of oils shows high values for oils, but grape seed oil and fenugreek oil have a higher level of chlorophylls (8.078 ppm) and carotene (56.78 ppm) (Table 3).

### 3.2. Antioxidant Activities

#### 3.2.1. DPPH Free Radical-Scavenging Assay

**Figure 1:**
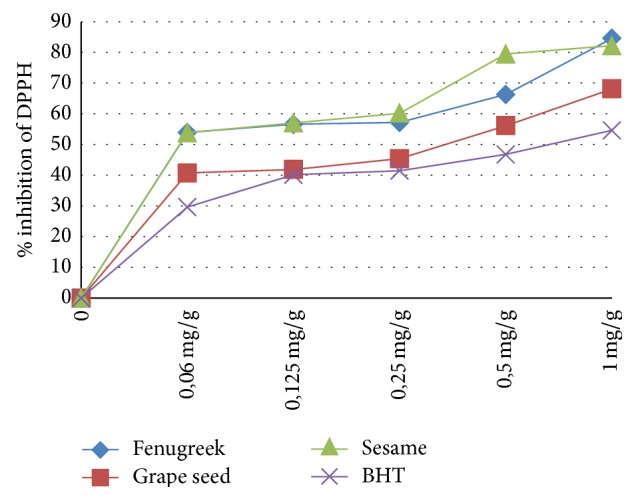
Activity of DPPH free radical scavenging.

The antioxidizing efficiency increases with the concentration of the oil (Figure 1). The three oils seem to have a potential antioxidant activity compared to the BHT activity. The percentages of inhibition are 68.12%, 84.59%, and 84.6% for grape seed oil, sesame oil, and fenugreek oil, respectively, and 54.6% for BHT at a concentration of 1 mg/g.

#### 3.2.2. Ferric Reducing Antioxidant Power FRAP

**Figure 2:**
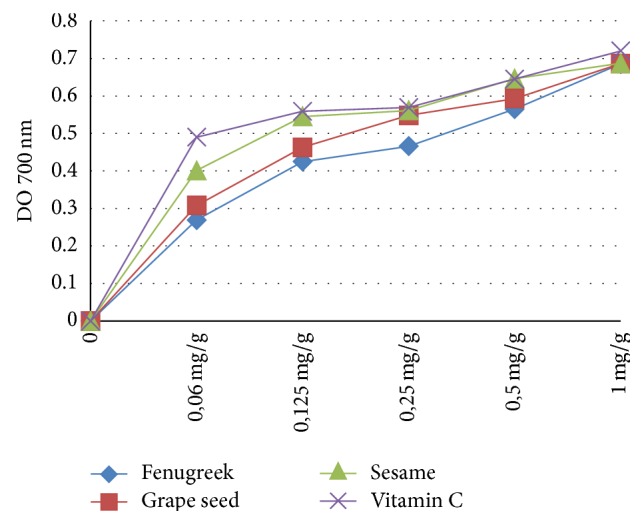
Ferric reducing power assay.

Figure 2 illustrates the reducing capacity of oils on iron III.

#### 3.2.3. *β*-Carotene Bleaching Assay

**Figure 3:**
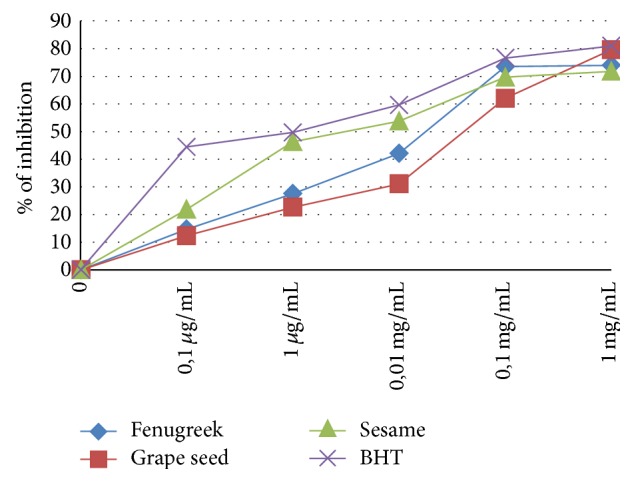
Activity of inhibition of bleaching of *β*-carotene.

Figure 3 demonstrates the *β*-carotene bleaching assays results.

### 3.3. Antibacterial Activity

#### 3.3.1. Diameter of Inhibition Zone

The antimicrobial activity of oils tested on different bacteria gram(+) and gram(−) was presented in Table 4.

#### 3.3.2. Determination of Minimum Inhibitory and Bactericidal Concentrations (MIC and MBC *μ*g/mL MH)

Table 5 points to the concentrations of MIC and MBC.

#### 3.3.3. Ratio = MBC/MIC

This ratio was used in order to verify the antimicrobial potential of oils, and the ratio MBC/MIC is required (Table 6).

### 3.4. Wound Healing Activity Test

The wound healing process was carried out by a chromatic study on the basis of the progressive changes in wound color during the different phases of cicatrization for each group: fenugreek oil, grape seed oil, sesame oil, “*CICAFLORA,*” and physiologic serum.

#### 3.4.1. Wound Healing Evaluation Parameters

Weights of Wistar rats were illustrated in Table 7. The statistical comparison of their average weight for the same group before and after treatment was not significant (*p* < 0.05).

#### 3.4.2. Chromatic Study

**Figure 4:**
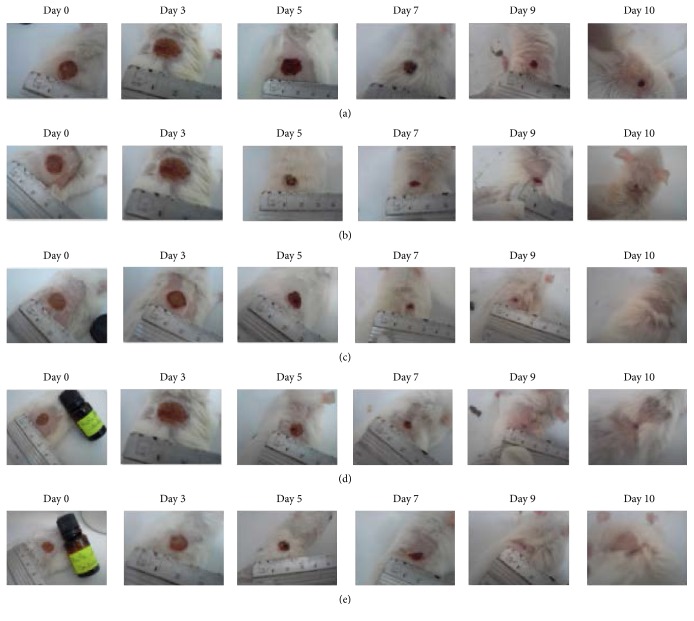
Visual observation of the wound healing experiment on days 0, 3, 5, 7, 9, and 10. (a) Untreated group, (b) group treated with “CICAFLORA,” (c) group treated with grape seed oil, (d) group treated with sesame oil, and (e) group treated with fenugreek oil.

Wound photography of the same group rats was illustrated in Figure 4. The chosen days (0/3/5/7/9 and 10) were corresponding to the wound induction day, inflammatory phase, granulation tissue formation, and reepithelialization, respectively.

The chromatic study of the wounds showed a similar coloration during the first three days. In fact, the bright red coloration observed on the wounding day reflected the color of the blood covering the underlying muscles after excision of the skin. This coloration became dark red on the second day which gave evidence of the formation of a blood clot. This clot enabled the blood coagulation. From the 3rd day, the blood clot was converted to a scab which retracted in the treated rats.

Towards the 7th day, the scabs allowed the apparition of a red coloration that corresponded to the tissue granulation with spread sides of the wound for the rats of the control group (untreated group).

**Figure 5:**
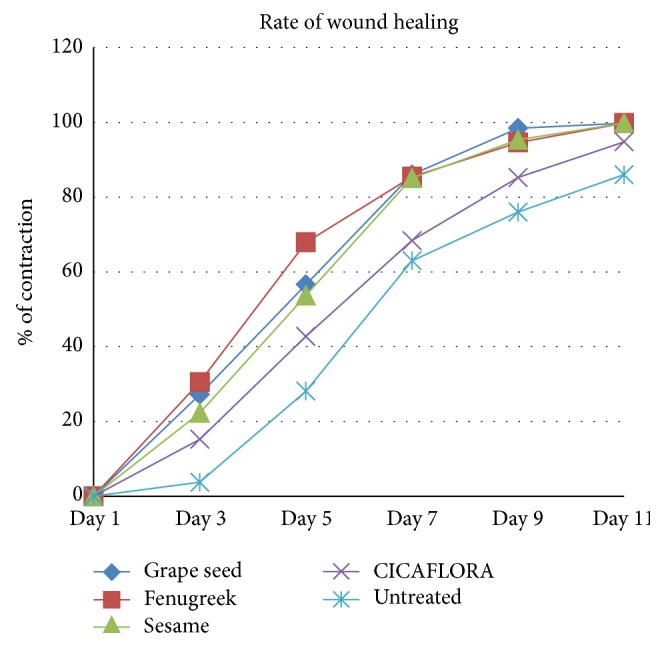
Rate of wound closure.

From the 10th day, these scabs in rats treated with oils and reference product began to fall to let a pinkish color appear that characterized an epithelialization ending after 11 days. The wound contracting ability of the oils and “*CICAFLORA*” was more significant than those of the control groups. The rate of wound closure by fenugreek oil, sesame oil, grape seed oil, and CICAFLORA and in the control group is illustrated in Table 8 and Figure 5. Their rates of wound closure at 11th day were, respectively, 99.84%, 99.83%, 99.84%, 94.82%, and 86.05%.

#### 3.4.3. Histological Examination

**Figure 6:**
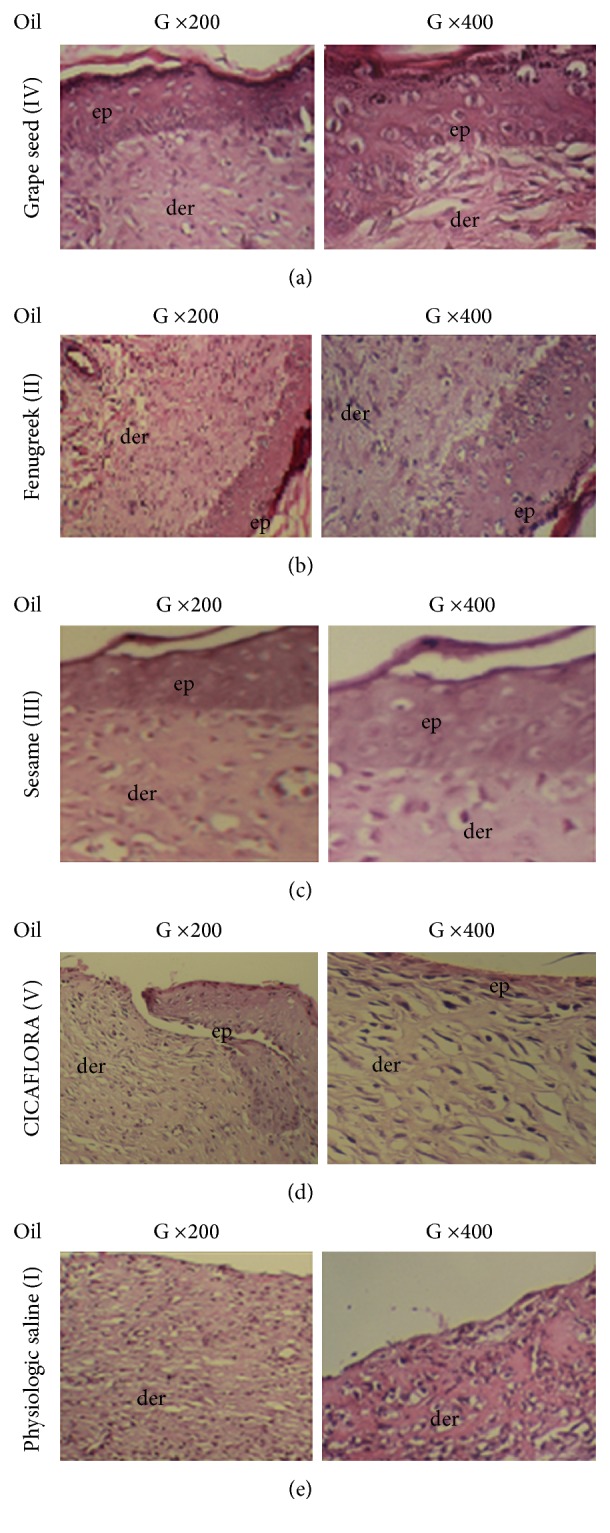
Histological investigation of treated and untreated biopsies: epidermal (ep) and dermal (der) architecture of wounds after the 11th day. (a) Group treated with grape seed oil, (b) group treated with fenugreek oil, (c) group treated with sesame oil, (d) group treated with “CICAFLORA,” and (e) untreated group (Gr200 and Gr400).

Epidermal regeneration covering over the wound surface treated by the fenugreek, grape seed, and sesame oils was colored by the hematoxylin-eosin to investigate the epithelium and tissue organization (Figure 6).

We noticed fibroconnective tissue regeneration in the reference biopsies and those of the three tested oils, without hairy adnexal or glandular structures. However, this tissue regeneration was lower in the biopsies of control rats. The microscopic examination of the scar zones handled by various oils highlighted an epidermic complete regeneration and well organized, which was considered normal. Also, the thickness of the epithelium was more important for the grape seed oil, sesame oil, and fenugreek oil than “*CICAFLORA*” cream.

## 4. Discussion

Various plants, essentially their oils, have been used to treat wounds. The literature presents several phytochemical constituents, various herbal formulations, and natural extracts from medicinal plants to the application for wound care. Some of those medicinal plants are traditionally used in folk medicine, including our plants, grape, fenugreek, and sesame, which are investigated in this study in order to explore their phytochemical compositions, to evaluate their wound healing effect, and to better understand their mechanism on wound healing. Consequently, topical application of the tested oils and the cicatrizing reference drug seems to accelerate the healing of wounds and the contraction of the skin borders to a fast recovery compared to the control. The best healing activity would be attributed to their physicochemical properties, antioxidant, and antibacterial activities.

So, oil acidity is the result of the degree of triacylglycerol distribution due to a lipolysis reaction, in which free fatty acids are formed. The acidity measured in the oil samples was low in the order of 0.2% for the fenugreek oil, 0.4% for the sesame oil, and 1.83% for the grape seed oil. All the studied oils were of acidic pH that promoted the inhibition capacity of bacteria growth and accelerated the wound healing process [23].

Peroxidation is a beginning to fat autoxidation, which is an inevitably slow phenomenon. The manipulation of oils and the manner of storage can reduce autooxidation effects. According to common regulations, the peroxide values of extra virgin olive oil must be under 20 Meq O_2_/kg [24], which concords with the results of the three oils. Those low peroxide values indicate that the tested oils were newly harvested and extracted and then stored in good conditions, suggesting that they kept a good quality over this work. The specific UV absorbance values at 232 nm, a primary oxidation indicator of oils, were 3.01 and the* K*_270_ values were lower. According to common standards, the UV absorbance at 232 nm for extra virgin olive oil must be under 2.5 [25]. These findings were concordant with the previous peroxide values. Both parameters, reflecting the degree of the oil autooxidation, can increase with the age of oils and their exposure to sunlight or high temperatures.

Tested oils contain a significant amount of unsaturated fatty acids that reach 89.37%, 82.5%, and 84.28% for grape seed oil, sesame oil, and fenugreek oil, respectively. The high level of polyunsaturated fatty acids can make it extremely susceptible to oxidation [26]. However, the oils were very stable due to the presence of a number of antioxidants like polyphenols, carotene, and chlorophylls. The antioxidants described in the present study can explain the lower values of the autooxidation parameters. In fact, several researches reported that many vegetable oils were an important natural source of carotenoids such as* Pistacia lentiscus* that presented a value ranging between 5.8 and 10.57 mg/kg oil [27], but our studied oils have substantially higher amounts especially in fenugreek and sesame oils 56.78 mg/kg and 15.24 mg/kg, respectively. Carotenoids are the most important source of vitamin A.

The autooxidation is the major cause of the deterioration of oil during the storage. It depends on several factors as the initial composition of the oil and the presence in minor compounds with pro- or antioxidizing activities like chlorophylls [28]. In our study, grape seed oil presented the highest value (8.078 mg/kg).

The autooxidation distorts edible oils by degradation of the essential fatty acids and consequently the reduction in the nutritional value and the formation of products of decomposition [29, 30]. The phenolic compounds in the three oils (197.56 ± 11.2 mg/kg), due to their antioxidizing activity, can contribute to the preservation of the quality of this one [31]. DPPH is a stable free radical which is reduced in the presence of an antioxidant, mostly by the phenolic compounds [32]. Indeed, the chemical structure of polyphenols enables them to trap this free radical by hydrogen transfer. Our results demonstrated that our tested oils reacted strongly against the DPPH radicals. This finding would be related to high concentrations of polyphenols in oils and explain their relative stability and lower autooxidation.

Other antioxidants in our studied oils described in literature can also contribute to their stability and to the wound healing phenomenon such as vitamin E and sterols [33].

Our study showed a total closure of the wounds treated by the oils and “*CICAFLORA*” after 11 days with an advanced tissue regeneration characterized by the presence of well-organized stratum of derma and epidermis in comparison with those of the control biopsies whose tissue neoformation was incomplete. According to literature, the natural contraction of wounds takes place by the 21st day [34]. This finding underlines the capacity of studied oils to accelerate the proliferation contributing to a fast recovery. The same healing period was also observed with two medicinal plant oils:* Cucurbita pepo. L* (Cucurbitaceae) and* Linum usitatissimum* (Linaceae) [35].

The chromatic study of the wounds showed a similar coloration during the first three days corresponding to the formation of a blood clot with debris of cell filling the breach in the course of the initial inflammatory phase. So, fibrin, once stabilized in blood clot, is a key element in the initial process of skin healing. It allows the recruitment of fibroblasts by chemotactic effect and stimulates the production of collagen [36]. When inflammatory cells arrive at the site of injury, they initiate a prolonged inflammatory phase that results in delayed deposition of matrix components, wound remodeling, and closure [37].

From the 3rd day, a proliferative phase has been triggered and characterized by the formation of granulation tissue, including angiogenesis, the migration of fibroblasts, and collagen synthesis [38]. In our study, from the 3rd day, the blood clot was converted to a scab which retracted in the treated rats. But an inflammatory reaction, manifested by an edema and oozing on wounds, seems more important in the rats of the control groups than those of all the three treated ones. Towards the 7th day, the scabs allowed the apparition of a red coloration that corresponded to the tissue granulation with spread sides of the wound for the rats of the control groups. In the treated groups, an important wound contraction was observed with an advanced reepithelization. The epithelial cells of the wound borders proliferated towards the center and led a complete wound closure towards the 11th day (Figure 5).

The phytoconstituents of the studied oils could explain the mechanisms of the skin wound healing process. So, their considerable amount of polyunsaturated fatty acids included oleic acid, linoleic acid, and linolenic acid. Linoleic acid, a precursor of arachidonic acid, is important in the inflammatory cascade (prostaglandins, thromboxanes, and leukotrienes) [39]. These substances act as inflammatory mediators and accelerate the inflammatory process. Thus, they increase local neovascularization, the remodeling of the extracellular matrix, migration, and fibroblastic cell differentiation [38], which accelerates the healing of wounds. Fatty acids have been reported to have the ability to reduce transepidermal water loss and increase skin hydration and supportive environment for accelerated skin wound healing [40].

In addition, the wound healing effect would also be attributed to a synergy between an antibacterial and antioxidant action observed in our study and described in the literature.

Generally, bacterial species have optimum moisture content closed to neutrality (6.5 < pH < 7.5). Habitually, bacteria need a pH ranging from 5.5 to 8.0 to grow; otherwise, there is a slowdown in their development activity, reaching a complete growth cessation at a pH under 4.5 or above 9.0. All the oils showed acidic pH that promoted the inhibition of bacterial growth and accelerated the wound healing process especially in the inflammation phase. Acidic pH contributed to the ideal environment for fibroblastic activity, cell migration, cell proliferation, and reorganization of collagen, which resulted in the stimulation of wound healing [41].

Polyphenols and carotenoids together with vitamin E and sterols demonstrated a beneficial effect on wound healing and collagen synthesis by preventing damaging effects of free radicals and ensuring the stability and integrity of biological membranes [42]. Palmieri et al. [43] also described how vitamin E has a humectant effect on skin wound scarring.

In addition, sterols are powerful compounds that can help to reduce systemic inflammation [44]. They can speed new skin growth by stimulating macrophages and increasing fibroblast and collagen production.

## 5. Conclusions

In conclusion, according to our experimental results, fenugreek, grape seed, and sesame oils proved to have a better activity on the wound healing compared to the “*CICAFLORA.*” This might be due to the synergistic effect of the phytoconstituents present in the oils. However it is necessary to initiate clinical trials on humans to confirm their efficacy in human pathology.

## Figures and Tables

**Table 1:** Values of phytochemical tests.

Oils	Phytochemical test				
	Acidity value	UV constants		Peroxide value	Saponification value
	%	*K*_232_	*K*_270_	Meq O_2_/Kg	170 < *x* < 255
Grape seed	1.83	3.0113	2.1710	12	175.32
Sesame	0.4	3.0103	1.1547	0.9	173.91
Fenugreek	0.2	3.0079	0.4373	11	172.32

**Table 2:** Fatty acids components.

Oil	Fatty acid				
	C16:0	C18:0	C18:1	C18:2	C18:3
	Palmitic acid%	Stearic acid%	Oleic acid% (Ω9)	Linoleic acid% (Ω6)	*α*-Linolenic acid% (Ω3)
Grape seed	6.69	3.57	19.75	69.33	0.29
Sesame	10.44	3.18	40.19	41.94	0.37
Fenugreek	8.36	3.60	19.19	37.71	27.38

**Table 3:** Polyphenols, chlorophylls, and carotene values.

Oils	Polyphenols	Chlorophylls	Carotene
	(ppm)	(ppm)	(ppm)
Grape seed	191.1	8.078	15.24
Sesame	191.3	0.005	3.98
Fenugreek	210.3	0.229	56.78

**Table 4:** Diameter of inhibition zone (mm).

Oils	Stains			
	Gram(+)		Gram(−)	
	*Bacillus subtilis*	*Staphylococcus aureus*	*Escherichia coli*	*Salmonella enteritidis*
	*JN 934392*	*ATCC 6538*	*ATCC 25922*	*ATCC 43972*
Grape seed	17 ± 1.0	—	11 ± 1.0	11 ± 1.0
Sesame	12 ± 0.5	—	—	—
Fenugreek	10 ± 1.0	—	—	—

**Table 5:** Concentration of bacteria (*μ*g/mL MH).

Oils	Stains							
	Gram(+)				Gram(−)			
	*Bacillus subtilis*		*Staphylococcus aureus*		*Escherichia coli*		*Salmonella enteritidis*	
	*JN 934392*		*ATCC 6538*		*ATCC 25922*		*ATCC 43972*	
	MIC	MBC	MIC	MBC	MIC	MBC	MIC	MBC
Grape seed	6.25	12.5	—		6.25	12.5	6.25	12.5
Sesame	6.25	25	—		—		—	
Fenugreek	3.125	25	—		—		—	

MIC: minimal inhibitory concentration, MBC: minimal bactericide concentration.

**Table 6:** Bactericide effect of tested oils.

Oils	Stains			
	Gram(+)		Gram(−)	
	*Bacillus subtilis*	*Staphylococcus aureus*	*Escherichia coli*	*Salmonella enteritidis*
	*JN 934392*	*ATCC 6538*	*ATCC 25922*	*ATCC 43972*
Grape seed	Bactericide	—	Bactericide	Bactericide
Sesame	Bacteriostatic	—	—	—
Fenugreek	Bacteriostatic	—	—	—

**Table 7:** Average weights before and after treatment with controls and oils.

Day	Average weight, untreated (g)	Average weight, CICAFLORA (g)	Average weight, grape seed oil (g)	Average weight, fenugreek (g)	Average weight, sesame (g)
Before treatment	182,5 ± 2.271	184.5 ± 2,271	176.5 ± 2.121	193,5 ± 2,121	172,33 ± 2,926
After treatment	183 ± 2.265	184.33 ± 2.559	176 ± 2.212	193 ± 2.265	171 ± 2.849
*p* value	0.25	0.085	0.25	0.25	0.66

Student's *t*-test was applied to detect and ascertain significant differences.

**Table 8:** Statistical study: median IQR and nonparametric tests.

	1	3	5	7	9	11
Grape seed	1.29 (1.12–1.38)	0.90 (0.84–1.12)	0.56 (0.56–0.60)	0.18 (0.15–0.18)	0.04 (0.02–0.06)	0.002 (0.002–0.003)
		** (**b^*∗*^ **)**	**(**b^*∗∗*^ **)**	**(**a^*∗∗*^, b^*∗∗*^ **)**	**(**a^*∗∗*^, b^*∗∗*^ **)**	**(**a^*∗∗*^, b^*∗∗*^ **)**
Fenugreek	1.38 (1.17–1.38)	0.94 (0.84–0.97)	0.42 (0.42–0.43)	0.18 (0.18–0.21)	0.08 (0.04–0.09)	0.003 (0.001–0.003)
		**(**a^*∗*^, b^*∗∗*^ **)**	**(**a^*∗∗*^, b^*∗∗*^ **)**	**(**a^*∗∗*^, b^*∗∗*^ **)**	**(**a^*∗∗*^, b^*∗∗*^ **)**	** (**a^*∗∗*^, b^*∗∗*^ **)**
Sesame	1.29 (1.03–1.31)	0.94 (0.94–1.12)	0.56 (0.56–0.84)	0.18 (0.16–0.21)	0.05 (0.023–0.094)	0.002 (0.001–0.003)
		**(**b^*∗*^ **)**	**(**b^*∗*^ **)**	**(**a^*∗∗*^, b^*∗∗*^ **)**	** (**a^*∗∗*^, b^*∗∗*^ **)**	** (**a^*∗∗*^, b^*∗∗*^ **)**
CICAFLORA	1.17 (1.00–1.50)	1.03 (0.96–1.12)	0.77 (0.56–0.77)	0.38 (0.37–0.41)	0.18 (0.17–0.19)	0.06 (0.06–0.07)
			**(**b^*∗*^ **)**	**(**b^*∗*^ **)**	**(**b^*∗∗*^ **)**	**(**b^*∗∗*^ **)**
Untreated	1.17 (1.15–1.41)	1.22 (0.99–1.38)	0.91 (0.81–0.96)	0.50 (0.38–0.50)	0.32 (0.27–0.32)	0.21 (0.11–0.21)

Nonparametric tests: Kruskal Wallis and Mann Whitney tests. Raw data shown with median IQR (*n* = 6) for each group.

^*∗*^ *p* < 0.05, ^*∗∗*^ *p* < 0.001. a: compared to CICAFLORA; b: compared to untreated.

## Additional Points

*Availability of Data and Materials*. The published and the software applications/tools were readily available to any scientist wishing to use them for noncommercial purposes, without restrictions.
